# Comparison of Wild and Field-Cultivated *Thymus satureioides*: Effects on Rhizosphere Properties, Essential-Oil Composition, and Biological Activities

**DOI:** 10.3390/plants15213398

**Published:** 2026-11-02

**Authors:** Meryem Youssfi, Oumaima Akachoud, Btissam Zoubi, Siham Houssayni, Anissa Lounès-Hadj Sahraoui, Frédéric Laruelle, Abderrazak Benkebboura, Toufic Elbeaino, Hafida Bouamama, Ahmed Qaddoury

**Affiliations:** 1 Laboratory of Sustainable Development and Health Research, Faculty of Sciences and Techniques, Cadi Ayyad University, Marrakech 40000, Morocco; akachoud.oumaima@gmail.com (O.A.); sihamhoussayni@gmail.com (S.H.); h.bouamama@uca.ac.ma (H.B.); 2 Laboratory of Agrobiotechnology and Bioengineering, Department of Biology, Faculty of Sciences and Techniques, Gueliz (FSTG) Cadi Ayyad University of Marrakech, Marrakech 40000, Morocco; btissamgcb@gmail.com (B.Z.); abderrazakbenkebboura@gmail.com (A.B.); qaddouryahmed2@gmail.com (A.Q.); 3 Unité de Chimie Environnementale et Interactions sur le Vivant, Université Littoral Côte d’Opale, UCEIV—UR n°4492, 62228 Calais, France; anissa.lounes@univ-littoral.fr (A.L.-H.S.); frederic.laruelle@univ-littoral.fr (F.L.); 4 Department of Integrated Pest Management of Fruit Trees and Vegetable Crops, Istituto Agronomico Mediterraneo di Bari, 70010 Bari, Italy

**Keywords:** PLFA, cultivation, EO, chemical composition, *Thymus satureioides* Coss., biological activities

## Abstract

*Thymus satureioides* Coss. is an endemic Moroccan medicinal and aromatic species of high socio-economic value, is increasingly threatened by overexploitation and continued reliance on wild harvesting. Sustainable cultivation could reduce pressure on natural populations, yet its effects on essential oil (EO) composition, bioactivity, and associated microbial diversity remain poorly understood. This study compared EO yield, chemical composition, antioxidant, antimicrobial, and nematicidal activities, and soil microbial diversity (PLFA) between wild (WT) and cultivated (CT) populations. Gas chromatography–MS analysis identified 18 and 24 compounds in WT-EO and CT-EO, respectively. Thymol and endo-borneol were the major constituents in both oils, with thymol occurring at higher levels in CT-EO (31.83%) than WT-EO (24.79%), while endo-borneol accounted for 15.61% and 16.37%, respectively. WT-EO was further characterized by the exclusive presence of camphor (0.28%). Despite the higher EO yield in CT plants, WT-EO exhibited stronger biological activities. WT-EO showed significantly higher antioxidant capacity, with lower IC_50_ values than CT-EO in TAC (0.01 vs. 0.02 mg mL^−1^), DPPH (0.30 vs. 0.60 mg mL^−1^), and ABTS (0.03 vs. 0.37 mg mL^−1^) assays. WT-EO also showed stronger antimicrobial activity, with lower MIC values against *E. coli* (2.5 vs. 3.8 mg mL^−1^), *Pseudomonas aeruginosa* (2.5 vs. 3.8 mg mL^−1^), and *Staphylococcus aureus* (0.04 vs. 0.50 mg mL^−1^). At the lowest tested concentration (5 µL mL^−1^), WT-EO induced significantly higher nematicidal activity than CT-EO, although both oils reached nearly complete juvenile mortality and egg-hatching inhibition at higher concentrations. WT soils also supported greater microbial biomass and a distinct PLFA-based microbial profile than CT soils (9.53 vs. 3.77 µg g^−1^ soil). These findings support cultivation as a sustainable production strategy while highlighting the importance of conserving WT populations as reservoirs of chemical and microbial diversity.

## 1. Introduction

The Lamiaceae family comprises numerous aromatic and medicinal plants with significant applications in the food, cosmetic, and pharmaceutical industries. Within this family, the genus *Thymus* L. includes approximately 215 species of herbaceous perennials and small shrubs known for their aromatic properties and diverse secondary metabolites, predominantly distributed in the Mediterranean region [1]. *Thymus* is highly represented in Morocco, with 21 reported species, including 13 endemics. Among them, *Thymus satureioides* Coss., locally known as “Zaater” or “Zaitra,” is an endemic species mainly found in the arid and semi-arid regions of the Anti-Atlas, Middle Atlas, and High Atlas Mountains. *T. satureioides* is rich in bioactive compounds such as thymol, carvacrol, and rosmarinic acid, which are associated with antioxidant, anti-inflammatory, and antimicrobial activities [2]. Previous studies have demonstrated that the essential oil composition of *T. satureioides* varies according to geographical origin, environmental conditions, and harvesting period, resulting in differences in its biological properties and potential applications [3,4]. Several investigations have highlighted the antimicrobial, antioxidant, and other pharmacological activities of *T. satureioides* EO and extracts, confirming its importance as a valuable medicinal species [2,5].

Economically, thyme is of great importance in Morocco, where it ranks second among the medicinal plants exported by the country. In 2023, the export value of thyme from Morocco was 6.16 M USD, with a total export volume of 1.05 K metric tons [6]. More recently, estimated thyme exports in Morocco reached around 2.94 million tons in 2021 and generated about USD 11.81 million in revenue [4]. These figures highlight the ecological and economic importance of *T. satureioides* as both a valuable medicinal resource and an important Moroccan export commodity. However, several wild thyme species, including *T. satureioides*, are currently recognized as being overharvested, and cultivation efforts have therefore been initiated. Nevertheless, most thyme production still relies on collection from approximately 300,000 hectares of Moroccan forests, placing considerable pressure on natural populations [4]. Additionally, climate-related pressures, particularly reduced rainfall, have contributed to the decline of wild aromatic plant populations in Morocco, and more than 90% of the country’s aromatic and medicinal plant (AMP) production still depends on wild harvesting, posing significant risks to biodiversity conservation and the long-term availability of these resources [7,8].

To address these challenges, cultivating AMP offers a sustainable strategy to reduce dependence on wild harvesting and could ensure a continuous and stable supply to meet market demand. However, there is a common misconception that cultivated plants may lack the “power or potential” of their wild counterparts, and this belief is not always justified. Several studies focusing specifically on *T. satureioides* have addressed this question. El-Bakkal et al. [9] compared wild and cultivated *T. pallidus* and *T. satureioides* over three consecutive years in Morocco and reported a slight decrease in EO yield together with reduced glandular trichome density on cultivated leaves, alongside changes in chemical composition and antioxidant/antimicrobial activity. Similarly, El Bouzidi et al. [10] compared wild and cultivated populations of three endemic Moroccan thyme species, including *T. satureioides*, and found quantitative differences in EO composition between wild and cultivated *T. satureioides*, although both origins retained strong antioxidant and antimicrobial activity. Conservation-oriented efforts have also included in vitro micropropagation of *T. satureioides* to relieve pressure on wild populations [11]. Together, these studies confirm that cultivation measurably alters the EO yield, chemistry, and bioactivity of *T. satureioides*, supporting the relevance of a wild-versus-cultivated comparison for this species.

More broadly, many studies have highlighted fluctuations in plant development, biochemistry, and bioactivity due to environmental changes, such as the transplantation from wild habitats to cultivated fields. Significant differences can also exist between microbial communities associated with plants in wild and cultivated environments, influenced not only by abiotic factors but also by agricultural practices [12]. Rhizospheric and endospheric microbes play a crucial role in stimulating plant metabolism and the biosynthesis of bioactive compounds, as well as modulating their responses to stress [13,14], and any changes in microbial diversity and population due to cultivation practices can affect the levels of secondary metabolites produced by the plant, potentially altering its medicinal properties or ecological interactions [15]. Cultivation practices can undeniably shape belowground microbial communities in myriad ways: soil amendments, irrigation schedules, monocropping, and reliance on agrochemicals substantially impact microscopic life in soil and roots [16], tending to modify naturally occurring microbial diversity and lessen complex ecological functions over time. As a result, cultivated environments with reduced biodiversity compared to wild ones are often found to have different biochemical profiles and bioactivities [17].

However, none of the studies conducted on *T. satureioides* to date, including those comparing wild and cultivated populations [9,10], has examined the soil environment underlying these chemical and biological changes. In particular, relationships among cultivation-induced changes in soil microbial functional groups, soil physicochemical characteristics, EO composition, and biological activities remain unexplored for this species, and nematicidal activity has not previously been assessed alongside antioxidant and antimicrobial properties. Therefore, the present study aimed to provide a comprehensive assessment of the effects of cultivation on *T. satureioides* by comparing wild and cultivated populations in terms of EO yield, chemical composition, and antioxidant, antimicrobial, and nematicidal activities, as well as soil physicochemical properties and microbial community structure based on phospholipid fatty acid (PLFA) analysis. To the best of our knowledge, this is the first study to integrate these complementary approaches to investigate how cultivation-associated changes in the rhizosphere may influence the quality and bioactivity of *T. satureioides* EO.

## 2. Results

### 2.1. Rhizospheric Soil Physicochemical Properties

Soil physicochemical properties differed markedly between wild (WT-soil) and cultivated (CT-soil) *T. satureioides* sites (Table 1). Although both soils were alkaline, CT-soil showed significantly higher organic matter (1.31% vs. 0.31%), limestone content (41.94% vs. 36.41%), electrical conductivity (113.16 vs. 46.06 us cm^−1^), and moisture levels (25.82% vs. 6.12%) than WT-soil.

Rhizospheric soils analysis reveals significant variations between cultivated and wild sites (Table 2). Compared to WT-soil, CT-soil displayed lower SiO_2_ (25.31% vs. 27.34%) and Na_2_O (0.23% vs. 2.60%) levels, but higher CaO (18.38% vs. 14.71%), Fe_2_O_3_, and Al_2_O_3_ contents. Conversely, K_2_O and P_2_O_5_ levels were similar. The total oxide content was slightly higher in CT-soil (79.57%) than in WT-soil (77.98%).

### 2.2. Phospholipid-Derived Fatty Acids (PLFA) Analysis:

**Figure 1:**
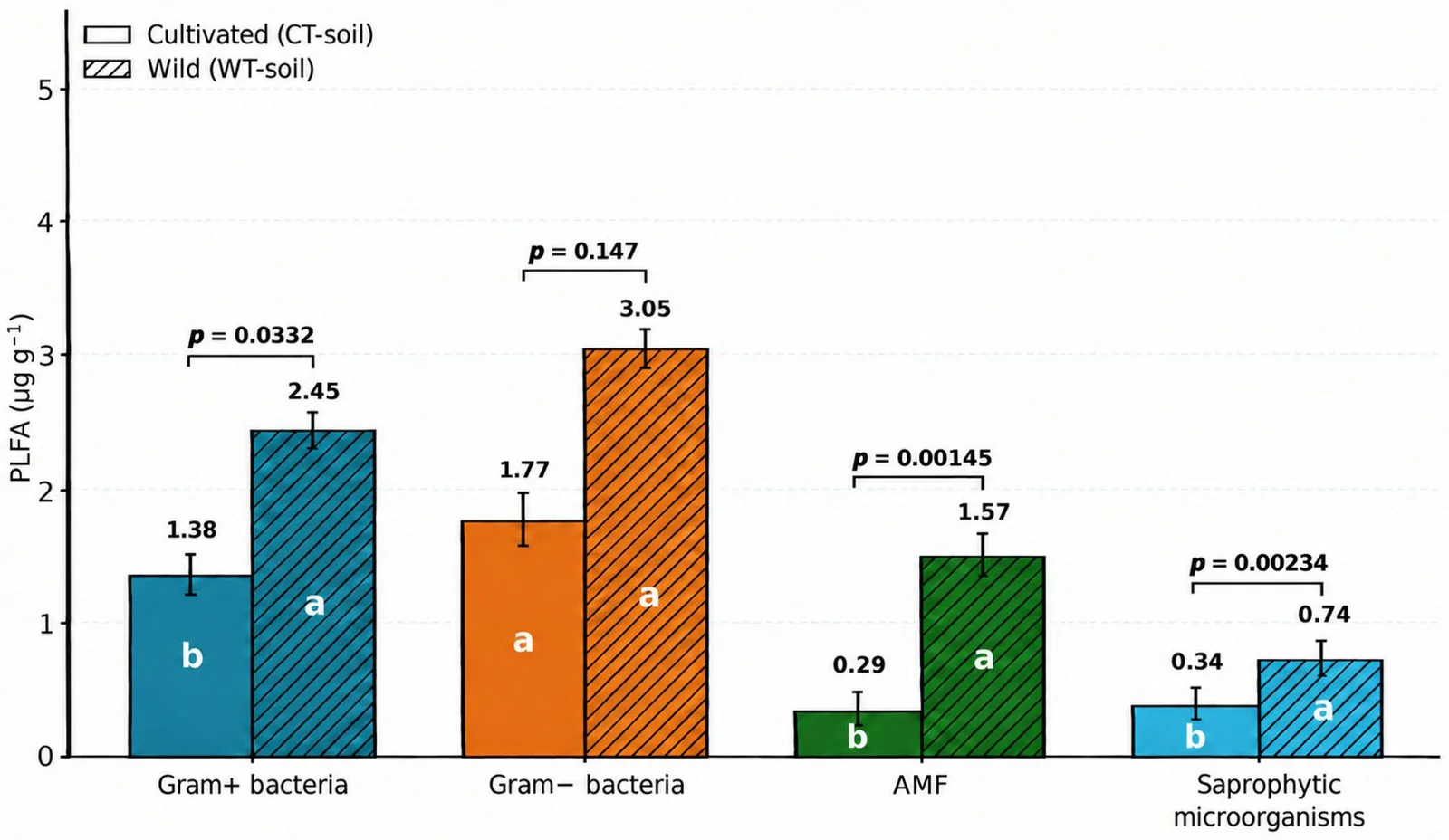
Soil phospholipid−derived fatty acids of wild and cultivated *T. satureiodes* sites. Different superscript letters (a, b) indicate statistically significant differences at *p* < 0.05.

PLFA analysis revealed a markedly higher total microbial biomass in WT-soil (9.53 µg g^−1^) than in CT-soil (3.77 µg g^−1^) (Figure 1). WT-soil displayed higher abundance of Gram-negative bacteria (4.80 vs. 1.77 µg g^−1^), Gram-positive bacteria (2.45 vs. 1.38 µg g^−1^), Arbuscular mycorrhizal fungi (AMF) (1.57 vs. 0.30 µg g^−1^), and other fungal groups (0.73 vs. 0.34 µg g^−1^).

### 2.3. EO Extraction and Phytochemical Profile Analysis

#### 2.3.1. EO Yield

Essential-oil yield was higher for the three individually extracted CT plants than for the three WT plants (1.60 ± 0.09% vs. 1.00 ± 0.12%, respectively; Table 3). The values describe variability among three plant-derived oils at each site; because each population was represented by one site, they do not establish a general cultivation effect.

Values are mean ± standard deviation for three independent plant-derived EO samples per site (*n* = 3 biological plant replicates). Plants were extracted separately before any pooling of EO for chemical profiling. The single wild site and single cultivated site do not permit site-level inference about cultivation.

#### 2.3.2. EO Composition

GC-MS analysis identified 18 compounds in WT-EO and 24 in CT-EO (Table 4). Monoterpene hydrocarbons occurred in similar proportions in CT-EO (28.09%) and WT-EO (30.61%), whereas sesquiterpene hydrocarbons were more abundant in CT-EO (5.80% vs. 0.80%). Qualitatively, the major constituents of CT-EO were thymol (31.83%), endo-borneol (15.61%), γ-terpinene (7.52%), camphene (6.13%), p-cymene (5.23%), and α-pinene (4.20%). In WT-EO, the predominant compounds were thymol (24.79%), endo-borneol (16.37%), α-terpineol (8.23%), γ-terpinene (7.39%), camphene (6.54%), and limonene (5.30%). Camphor was exclusive to WT-EO, whereas β-myrcene, α-terpinolene, terpinen-4-ol, and caryophyllene were only detected in CT-EO (Figures S1 and S2).

### 2.4. Biological Activities

#### 2.4.1. Antioxidant Activity

**Figure 2:**
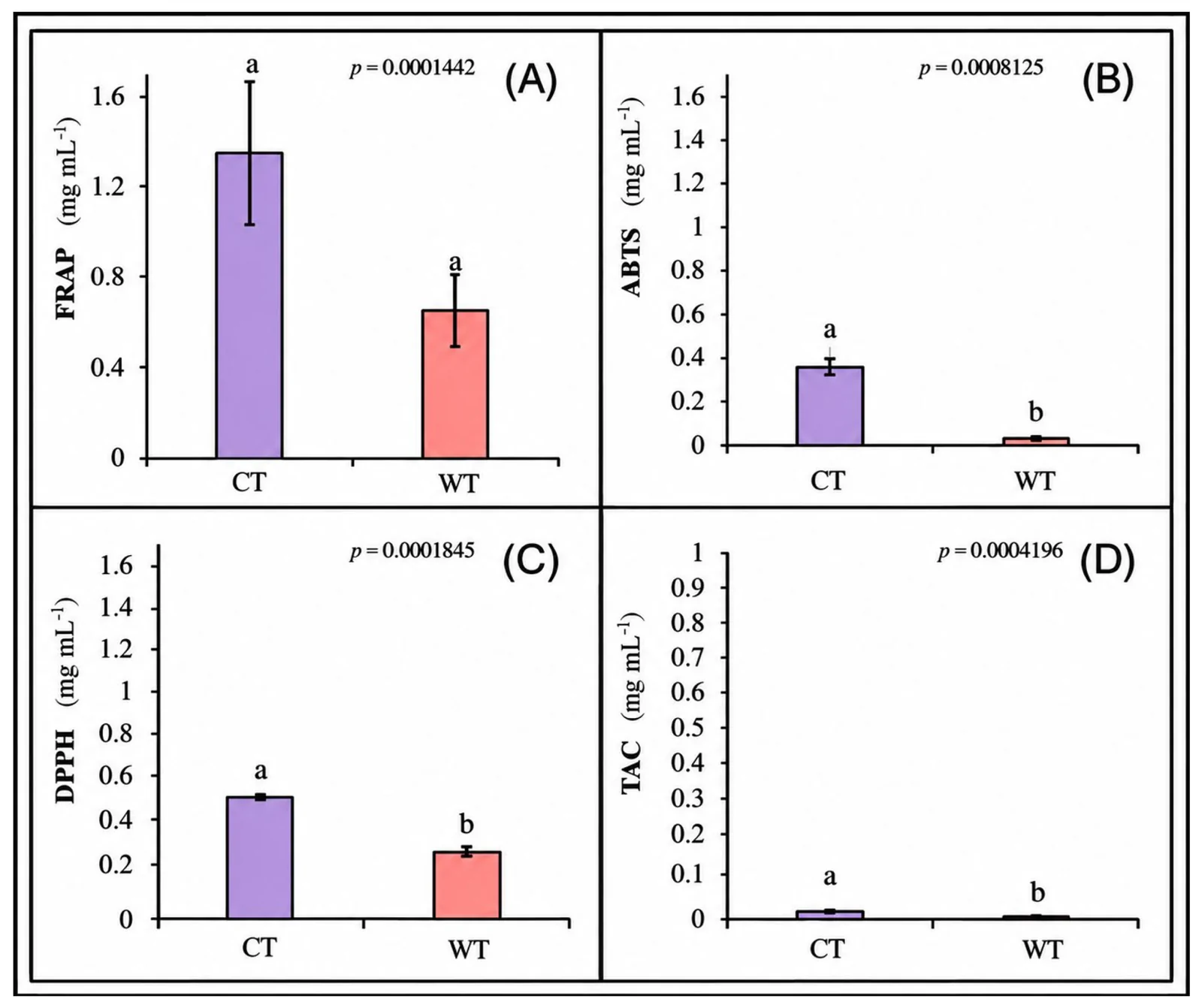
Antioxidant activity of EO from cultivated (CT) and wild (WT) *T. satureioides* measured by (**A**) FRAP, (**B**) ABTS, (**C**) DPPH, and (**D**) TAC assays. Values are presented as mean ± standard error (*n* = 3). Different lowercase letters above bars indicate significant differences among treatments according to one-way ANOVA followed by Tukey’s HSD test (*p* < 0.05).

Both EO displayed antioxidant activity, with WT-EO exhibiting significantly higher activity than CT-EO (Figure 2). This superior performance was evidenced by lower IC_50_ values in TAC (0.01 vs. 0.02 mg mL^−1^), DPPH (0.30 vs. 0.60 mg mL^−1^), and ABTS (0.03 vs. 0.37 mg mL^−1^) assays.

#### 2.4.2. Antimicrobial Activity

Both EOs displayed antimicrobial activity against all tested pathogens, with WT demonstrating significantly higher efficacy than CT (Table 5). WT-EO exhibited lower MIC values against *Escherichia coli* (2.5 mg mL^−1^), *Pseudomonas aeruginosa* (2.5 mg mL^−1^), *Enterococcus hirae* (0.05 mg mL^−1^), and *Staphylococcus aureus* (0.04 mg mL^−1^) compared to CT, with MIC values of 3,8 mg mL^−1^, 3.8 mg mL^−1^, 0.10 mg mL^−1^, and 0.50 mg mL^−1^) respectively. Similarly, WT-EO exhibited superior anti-fungal activity against *Fusarium oxysporum*, with lower MIC (0.155 mg mL^−1^) than CT-EO (0.187 mg mL^−1^).

#### 2.4.3. Nematicidal Activity

**Figure 3:**
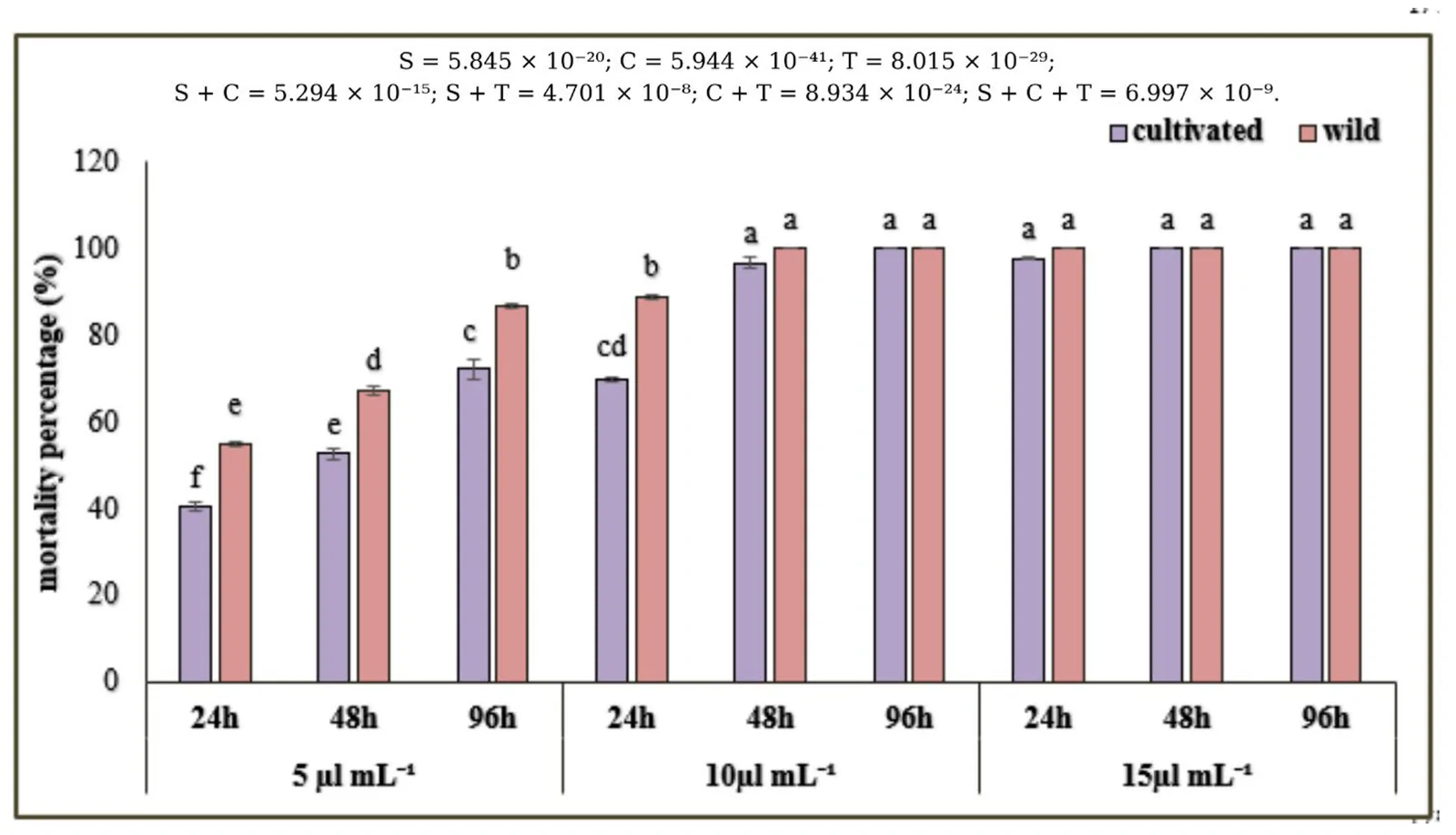
Mortality rate of *Meloidogyne javanica* juveniles (J2) induced by EO from wild (WT) and cultivated (CT) *T. satureioides* at different concentrations and exposure times. S: EO source; C: concentration; T: exposure time. Different lowercase letters indicate significant differences among treatments (*p* < 0.05).

**Figure 4:**
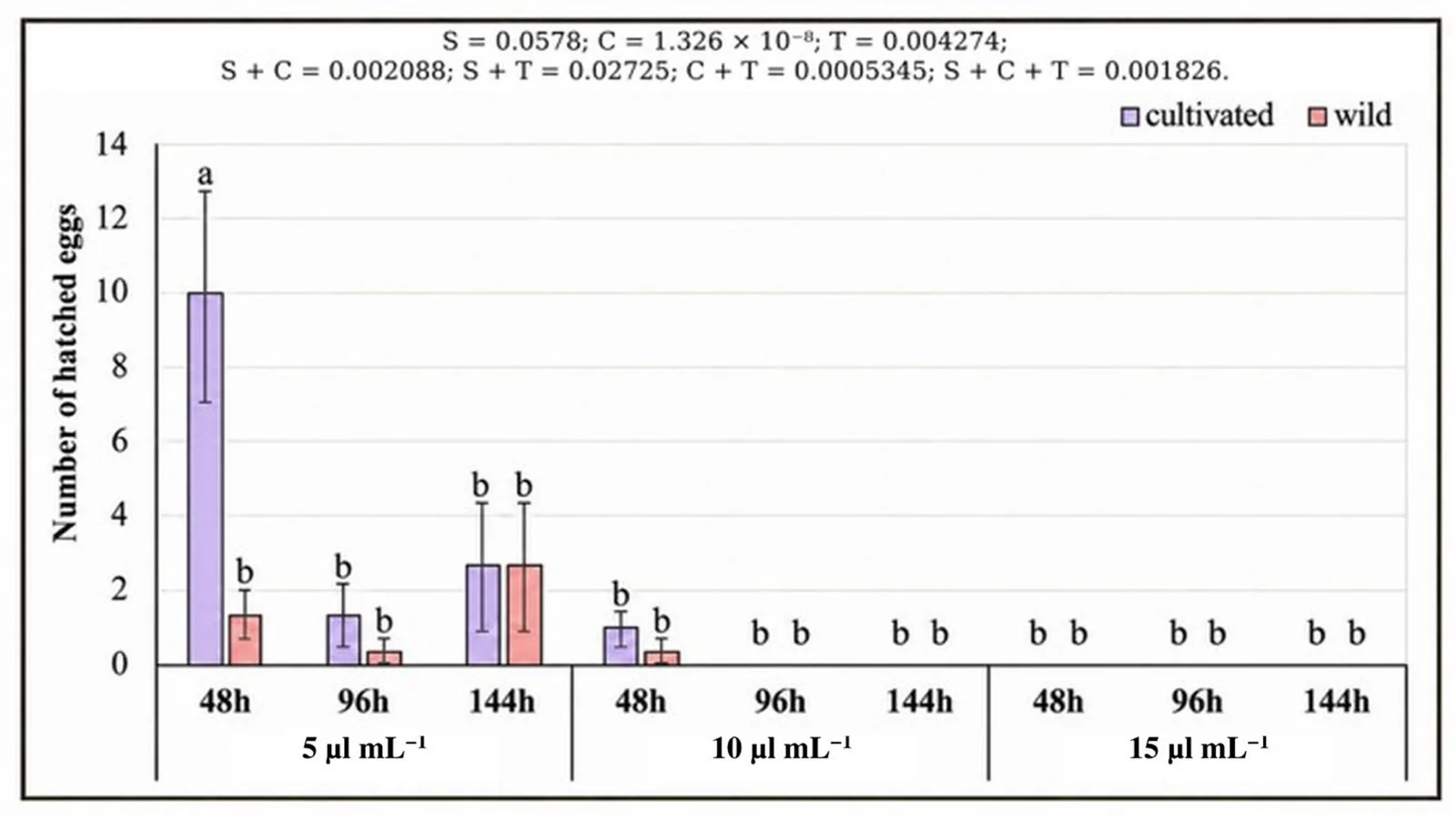
Effect of EO from wild (WT) and cultivated (CT) *T. satureioides Meloidogyne javanica* egg hatching at different concentrations and incubation periods. S: EO source; C: concentration; T: exposure time. Different lowercase letters indicate significant differences among treatments (*p* < 0.05).

Both EOs exhibited dose- and time-dependent nematicidal activity. At 5 µL mL^−1^, WT-EO induced significantly higher juvenile mortality (Figure 3) and egg-hatching inhibition (Figure 4) than CT-EO. However, at 10 and 15 µL mL^−1^, both oils achieved nearly 100% juvenile mortality and maximum egg-hatching suppression, showing no significant difference between treatments.

### 2.5. Heat Map Analysis

**Figure 5:**
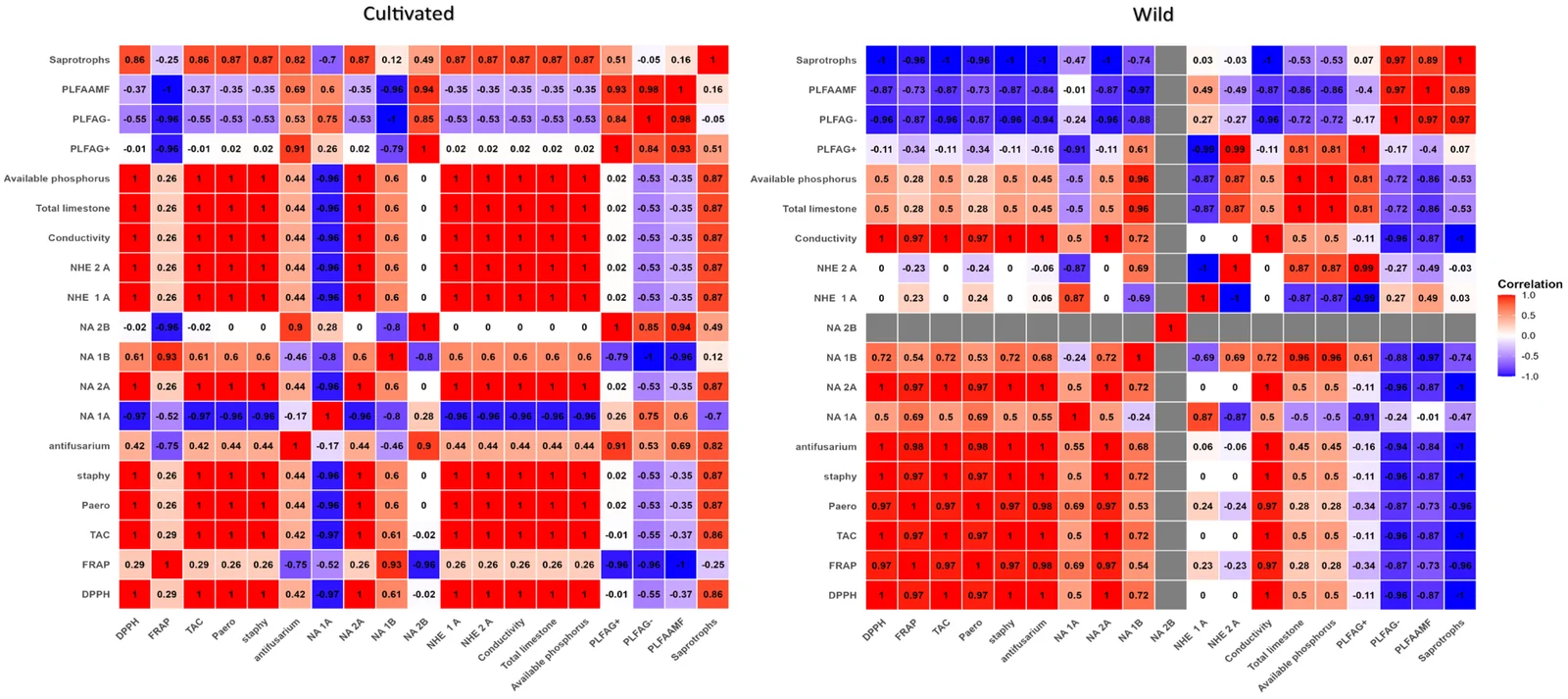
Heatmap correlation analysis between EO yield, biological activities, and soil microbial parameters in wild and cultivated *T. satureioides*. The heatmap represents Pearson correlation coefficients (r) among measured variables, with positive correlations indicating variables that increase together and negative correlations indicating opposite trends. The analysis was used to visualize associations between EO characteristics, biological activities, and PLFA-derived microbial parameters. Paero: *Pseudomonas aeruginosa*; Staphy: *Staphylococcus aureus*; NA1A and NA1B: nematicidal activity at 5 µL mL^−1^ after 24 and 48 h of exposure, respectively; NA2A and NA2B: nematicidal activity at 10 µL mL^−1^ after 24 and 48 h of exposure, respectively; NHE1A and NHE1B: number of hatched eggs at 5 µL mL^−1^ after 24 and 48 h of exposure, respectively; NHE2A and NHE2B: number of hatched eggs at 10 µL mL^−1^ after 24 and 48 h of exposure, respectively; PLFAG+ and PLFAG−: Gram-positive and Gram-negative bacterial biomass, respectively; PLFAAMF: arbuscular mycorrhizal fungal biomass.

Heatmap correlation analysis was performed to visualize relationships among EO yield, biological activities, and soil microbial parameters in wild and cultivated samples (Figure 5). In WT samples, strong positive correlations (r > 0.90) were observed among several biological activity parameters, including antibacterial activity against *Staphylococcus aureus* and *Pseudomonas aeruginosa*, antifungal activity against *Fusarium*, and antioxidant assays (DPPH, TAC, and FRAP). In contrast, CT samples showed a more heterogeneous correlation pattern, with weaker or more variable relationships among antioxidant, antimicrobial, nematicidal activities, and soil microbial parameters. Overall, the heatmap highlights differences in the correlation patterns between wild and cultivated samples and serves as an exploratory visualization of associations among the measured variables.

## 3. Discussion

Different biological activities were observed between the wild and cultivated *T. satureioides* populations studied, coinciding with differences in their chemical profiles and contrasting environmental and soil conditions. WT-EO exhibited higher antioxidant, antimicrobial, and nematicidal activities than CT-EO. These findings differ from a previous study on *T. satureioides*, where antioxidant activity was not significantly affected by cultivation, although antimicrobial responses showed some variation [18]. Such discrepancies may arise from differences in EO composition, geographical origin, extraction methods, microbial susceptibility, and the complex interactions among individual constituents, including possible synergistic or antagonistic effects between major and minor compounds [18,19].

The variation in the relative abundance of major terpenoids between WT-EO and CT-EO may partly explain these differences in bioactivity. Thymol, the predominant compound in both EOs, was present at a higher relative abundance in CT-EO (31.83%) than in WT-EO (24.79%). Sbayou et al. [20] similarly reported borneol and thymol as the predominant constituents of *T. satureioides* EO, although their relative abundances differed from those observed here; a more recent analysis of 40 cultivated accessions from different Moroccan regions also identified borneol and thymol as dominant constituents while revealing pronounced chemotypic variation among populations, underscoring the considerable chemical polymorphism of this species [21]. Thymol is widely recognized for antioxidant and antimicrobial activity through radical scavenging and membrane-disrupting mechanisms [22,23]. However, its higher content in CT-EO did not correspond to stronger biological activity, indicating that EO bioactivity depends on the overall chemical profile and interactions among multiple constituents rather than on a single major compound. Other oxygenated monoterpenes that differed in abundance between WT-EO and CT-EO, particularly endo-borneol and α-terpineol, have previously been associated with antioxidant and antimicrobial activities [24,25,26], while thymol, borneol derivatives, and terpineol compounds have also been linked to nematicidal activity against plant-parasitic nematodes [27,28]; differences in the proportions of these compounds may therefore have contributed to the stronger biological responses observed for WT-EO.

Beyond these major constituents, minor monoterpenes and sesquiterpenoids may also contribute to EO bioactivity. WT-EO contained higher levels or unique detection of compounds such as T-muurolol, D-limonene, and caryophyllene oxide, which may enhance activity through interactions with major constituents: muurolene has demonstrated antimicrobial effects against *E. coli* and other microbial strains [29,30], caryophyllene oxide exhibits antimicrobial and antioxidant potential [31], and D-limonene, cis-ocimene, and T-muurolol have been associated with antibacterial, antioxidant, and anti-inflammatory effects [32,33]. Together, these observations suggest that the stronger biological activity of WT-EO likely results from the combined contribution and possible interactions of multiple chemical constituents rather than from individual compounds alone. This is consistent with the MIC values obtained, which were comparable to those previously reported for other *Thymus* EOs, although variation among studies is commonly observed due to differences in EO chemotype, geographical origin, extraction method, microbial susceptibility, and assay conditions [34,35]. Thymol and carvacrol-rich *Thymus* oils generally show stronger inhibitory effects against Gram-positive bacteria, whereas higher MIC values are often reported against Gram-negative strains [36,37]. This difference is attributed not only to the outer membrane, whose lipopolysaccharide layer and porins restrict the diffusion of hydrophobic terpenoids (Nikaido, 2003; Burt, 2004) [38,39], but also to active efflux systems such as AcrAB–TolC in *E. coli* and MexAB–OprM in *P. aeruginosa*, which extrude terpenoid components and act in synergy with low membrane permeability [40,41]. The poor aqueous solubility and volatility of EO constituents, and their interaction with the solvent and medium, may further reduce their effective concentration in broth [39,42]. Nevertheless, this barrier is not absolute, as thymol and carvacrol can disintegrate the outer membrane of Gram-negative bacteria [43]. As permeability and efflux were not assessed here, these explanations remain hypotheses.

These chemical differences are rooted in biosynthesis and abiotic conditions. The diversity and abundance of terpenoid compounds in EO are determined by complex biosynthetic processes involving the MVA and MEP pathways, which supply precursors for monoterpene and sesquiterpene formation via terpene synthases [44,45]. Regulation of these pathways is influenced by genetic and environmental factors, including light, nutrient availability, and altitude, which can modify secondary metabolism and contribute to chemical variability among plant populations [46,47]; such metabolic and ecological factors may partly explain the differences in terpenoid composition observed here between wild and cultivated *T. satureioides*. Soil chemical composition and abiotic factors are one such source of variation, as they can affect enzymatic processes involved in secondary metabolite biosynthesis and influence chemotype distribution and compound proportions [48,49]. Nutrient availability, salinity, soil texture, pH, and water availability have all been reported to modify phytochemical accumulation by affecting plant metabolism [50,51]. In the present study, cultivated soils exhibited higher nutrient availability than wild soils, whereas wild soils showed higher microbial biomass and functional group signals, a contrast suggesting that greater nutrient availability alone does not necessarily translate into increased EO activity. According to the carbon–nutrient balance hypothesis, nutrient limitation may instead promote the allocation of carbon resources toward secondary metabolites, including terpenes, when growth is constrained by limited nutrient availability [52,53]. This is consistent with several reports linking soil characteristics to EO composition in *Thymus* species: organic matter content and electrical conductivity influenced EO synthesis in *T. kotschyanus* [54], chemotype distribution in *T. pulegioides* has been associated with soil characteristics [55], and EO yield and composition in T. vulgaris have been related to nitrogen, phosphorus, and potassium availability [56]. However, soil nutrients can have both positive and negative effects depending on the metabolite considered; for example, potassium and phosphorus have been reported to decrease caryophyllene oxide synthesis in *Ocimum basilicum* EO [57], indicating that soil conditions regulate EO composition through complex interactions rather than a simple dose-dependent increase or decrease.

Beyond chemistry and abiotic soil properties, the two sites also differed markedly in soil microbial patterns, which may represent a further contributing factor. Wild soils exhibited higher PLFA-derived microbial biomass, with significantly higher signals associated with Gram-positive and Gram-negative bacteria, arbuscular mycorrhizal fungi (AMF), and saprotrophic microorganisms, compared with cultivated soils. This is consistent with previous studies indicating that reduced anthropogenic disturbance and lower agricultural pressure favor the maintenance of diverse microbial communities, including AMF and saprotrophic microorganisms [58,59]. The higher microbial biomass and greater representation of these functional groups in wild soils may reflect enhanced microbial functions related to nutrient cycling, organic matter transformation, soil structure maintenance, and plant–microbe interactions, whereas agricultural practices such as tillage and chemical inputs have been reported to modify microbial community structure and reduce sensitive microbial groups such as AMF through changes in soil organic matter and nutrient availability [60,61].

Several site- and management-related factors plausibly explain these contrasting microbial patterns. The cultivated plot remained fallow and vegetation-free for at least five years before planting, likely depleting AMF propagules and other rhizosphere-associated microorganisms; since the cultivated plants were only two years old at sampling, this period was probably insufficient for full microbial recolonization. In addition, the higher phosphorus availability in cultivated soils (1.94 vs. 1.11 g kg^−1^; Table 1), resulting from farmyard manure amendment, may have further suppressed AMF biomass, as plants reduce carbon allocation to mycorrhizal symbionts when phosphorus is readily available [62,63]. The comparatively high relative standard deviation observed in cultivated soils likely reflects spatial variability introduced by drip irrigation, which creates discrete wetting zones rather than uniform moisture distribution, increasing plant-to-plant variability among the five sampled individuals [64]. Notably, genetic background and altitude-related effects can largely be excluded as confounding factors, since the cultivated plants were propagated from transplants of the same wild source population and the two sites differ in altitude by less than 50 m; the sites nonetheless still differ in fallow history, irrigation regime, plant age, and occasional manure amendment, of which soil microbial community composition is only one component.

These soil microbial differences may in turn connect to plant secondary metabolism through several known mechanisms. Plant-associated microorganisms can influence secondary metabolism by modulating plant growth, stress responses, nutrient acquisition, and signaling pathways involved in metabolite production [65,66]. For instance, Piriformospora indica and *Azotobacter chroococcum* have been reported to enhance artemisinin accumulation in Artemisia species [67], plant–microbiome interactions in *Salvia miltiorrhiza* have been associated with altered tanshinone accumulation [68,69], and microbiome interactions in Echinacea purpurea have been linked to changes in volatile organic compounds, phenylpropanoids, and alkamides [70,71]. These studies support the possibility that differences in soil microbial conditions contribute to variation in plant secondary metabolism, and suggest that the higher PLFA-derived microbial biomass and greater abundance of bacterial, AMF, and saprotrophic markers in wild soils reflect a belowground environment distinct enough to have influenced EO composition, potentially via effects on nutrient cycling, plant stress responses, or plant–microbe signaling, and thereby contributed to the contrasting chemical and biological profiles of WT-EO and CT-EO.

These interpretations nonetheless warrant caution. PLFA analysis provides information on microbial biomass and broad functional groups rather than taxonomic identification; the observed differences should therefore not be interpreted as changes in specific microbial taxa or as direct evidence that particular microorganisms regulate EO biosynthesis. Similarly, the correlations depicted in the heatmap (Figure 5) between microbial parameters, EO yield, and biological activities should be read as descriptive associations rather than evidence of a causal link. Because the wild and cultivated sites differ in multiple environmental and management factors beyond microbial community composition, including land-use history, plant age, and irrigation regime, two soil environments shaped by different management histories would be expected to diverge in microbiome composition regardless of any functional role in secondary metabolism. The heatmap correlations therefore reflect co-variation among site- and management-associated parameters rather than a demonstrated causal link between microbial activity and EO biosynthesis, and controlled experiments involving microbial manipulation, soil transplantation, and molecular characterization of microbial communities will be required to establish causality.

A further limitation is that the wild and cultivated populations were each represented by a single site, so the observed differences cannot be unambiguously attributed to cultivation itself and may partly reflect microclimate, soil history, or other site-specific factors. Sampling across five randomly selected plants per site, with biological and technical replication, supports the reliability of the within-comparison estimates but does not resolve this site-level confound. These findings should therefore be interpreted as a comparison between two specific populations, and future studies incorporating multiple independent wild and cultivated sites are needed to establish a generalizable cultivation effect.

Overall, our results indicate that the wild and cultivated sites studied differed in soil environment, and that these differences were associated with distinct EO composition and biological activity in *T. satureioides*. The higher microbial biomass and distinct PLFA functional patterns observed in wild soils, together with differences in soil nutrient availability, offer a plausible ecological explanation for the distinct terpenoid profile and stronger biological activity of WT-EO. However, these relationships remain correlative, and future studies combining microbial community sequencing, controlled inoculation experiments, and metabolomic approaches are needed to determine whether specific microbial groups directly influence secondary metabolite production.

## 4. Materials and Methods

### 4.1. Plant and Soil Materials and Site Description

Aerial parts and rhizospheric soil samples of wild (WT, WT-Soil) and cultivated (CT, CT-Soil) *T. satureioides* were collected from Sidi Rahal, Marrakech, Morocco, (830 m altitude; N 31°40′7″, W 7°21′54″). The region features a hot semi-arid climate, with mean temperatures 10–28 °C (extremes −1 °C to 44 °C) and average annual rainfall of ~387 mm. The WT population was located on a low hill at 830 m altitude, whereas the CT population was established nearby with a 50 m elevation difference, ensuring identical ecological and climatic conditions. The CT plot had remained fallow and vegetation-free for at least five years before planting. CT plants were propagated from local WT cuttings and planted at a density of approximately ~6 plants m^−2^, with 50 cm inter-row spacing under drip irrigation. The cultivated plants were two years old at the time of sampling and were managed under low-input conditions, receiving only occasional applications of composted farmyard manure, with no synthetic fertilizers or pesticides applied. Botanical identification was confirmed by Prof. Ahmed Ouhammou, head of the Regional Herbarium MARK, where a voucher specimen of the studied plant material was deposited (voucher No. TS10011). The collected material was identified as *T. satureioides* Coss. Identification was performed at the species level, and no subspecies assignment was conducted in the present study.

At full flowering (May–June), fine roots (~5 g) and rhizospheric soil (2 kg) were sampled from three individual plants at a depth of 20–40 cm. Aerial parts were also collected from three individual plants, air-dried under shade separately per plant, and used to constitute three independent EO samples (Section 4.4.1). Soil was sieved (2 mm mesh) to remove debris. Fine roots and soil subsamples were stored at −20 °C for phospholipid fatty acid (PLFA) analysis, while the remaining soil was kept at room temperature for further physicochemical and biological analysis.

### 4.2. Physicochemical Soil Properties

Soil pH was measured in a 1:1 soil-to-water suspension [72] using a pH meter, and electrical conductivity was determined using a conductivity meter. Available phosphorus (P) was quantified using the Olsen method [73], and total nitrogen (N) was determined following the Kjeldahl procedure [74]. Potassium (K), calcium, and sodium concentrations were measured using a flame photometer [75]. Soil organic matter and organic carbon contents were determined by the Walkley-Black titration method [76]. Soil mineral composition was assessed by X-ray analysis technique using an Epsilon 4 model (Malvern Panalytical, Almelo, The Netherlands).

All physicochemical analyses were performed on each of the three soil samples collected per population (*n* = 3 biological replicates), with three technical replicates per sample.

### 4.3. Phospholipids Fatty Acids (PLFAs) Analysis

Soil PLFAs were extracted from 3 g of dried, sieved soil following Frostegård et al. [77], adapted from the Bligh and Dyer protocol. Lipids were extracted using a single-phase chloroform/methanol/citrate buffer mixture (1:2:0.8, *v*/*v*/*v*). After centrifugation, the pellet was re-extracted, and the combined supernatants were phase-separated overnight with chloroform and citrate buffer. The recovered organic phase was added with 20 µL of methyl nonadecanoate (19:0; 0.5 µg µL^−1^) as an internal standard, then evaporated under nitrogen N_2_ at 40 °C.

Lipids were fractionated on SPE silica columns into neutral lipids, glycolipids, and phospholipids using chloroform, acetone, and methanol, respectively. The phospholipid fraction was converted to fatty acid methyl esters (FAMEs) by mild alkaline methanolysis. FAMEs were extracted with hexane/chloroform (4:1, *v*/*v*) mixture, acetic acid, and water, before being evaporated under nitrogen and stored at −20 °C.

PLFAs were analyzed by GC-MS (70 eV electron impact) with ion source and transfer line at 250 °C and 280 °C, respectively. Fatty acids were identified using the NIST11 library and a commercial FAME mixture (C4–C24:1, Sigma-Aldrich, St. Louis, MO, USA), and quantified relative to the internal standard.

Bacterial biomass was estimated using the sum of PLFAs: i14:0, i15:0, a15:0, 15:0, i16:0, 10Me16:0, i17:0, a17:0, cy17:0, 17:0, br18, 10Me17:0, 18:1ω7, 10Me18:0, and cy19:0 [78], while fungal biomass was evaluated using the PLFA 18:2ω6,9.

For PLFA analysis, three independent rhizospheric soil samples were collected from each population (WT-soil and CT-soil), and each sample was processed separately for lipid extraction and phospholipid fatty acid (PLFA) determination. Microbial biomass and functional group abundances were therefore obtained from three independent biological replicates per population (*n* = 3).

### 4.4. EO Extraction and Phytochemical Identification

#### 4.4.1. Hydrodistillation Process

Shade-dried aerial parts of *T. satureioides* were used for EO extraction. For each independent extraction, 40 g of dried aerial material was subjected to hydrodistillation for 3 h in a 500 mL flask using a Clevenger-type apparatus. The hydrodistillation procedure was independently repeated three times, using a separate 40-g portion for each extraction, resulting in three independent EO samples (*n* = 3). The volatile compounds were condensed using a water-cooled system (~10 °C), and the recovered EO was separated from the aqueous phase, dried over anhydrous sodium sulfate, and transferred to hermetically sealed amber-glass vials. The EO samples were stored at 4 °C until further analysis to minimize oxidation and volatilization.

#### 4.4.2. EO Composition Analysis

EO samples were diluted in ethyl acetate (1:200, *v*/*v*) and analyzed using a gas chromatography–mass spectrometry system (GC-MS QP 2010 Ultra, Shimadzu, Kyoto, Japan). The system was equipped with a single quadrupole mass spectrometer and a flame ionization detector (FID). Helium was used as the carrier gas at a constant linear velocity of 60 cm s^−1^. A volume of 0.2 μL of diluted EO was injected in split mode (1:10) at 260 °C onto a ZB-5MS capillary column (5% phenyl–95% dimethylpolysiloxane; 10 m × 0.10 mm i.d., 0.10 μm film thickness; Phenomenex, Le Pecq, France). The oven temperature was programmed from 60 °C (held for 2 min) to 280 °C (held for 1 min) at a rate of 40 °C min^−1^. The mass spectrometer was operated in electron ionization mode at 70 eV, with an interface temperature of 280 °C and a mass acquisition range of 35–350 *m*/*z*.

Compounds were identified by comparing their mass spectra with those available in the NIST mass spectral library and by comparing their calculated retention indices (Kovats indices) with published values and entries from the Pherobase database. Retention indices were determined using the retention times of n-alkane standards analyzed under the same chromatographic conditions. The relative abundance of each compound was calculated from GC-FID peak areas without correction factors and expressed as a percentage of the total chromatographic area.

Essential oils independently extracted from three individual plants of each population (WT and CT) were analyzed separately by GC–MS. Each plant-derived essential oil represented an independent biological replicate (*n* = 3 per population). The relative abundance of each identified compound was calculated from the corresponding chromatographic peak areas and expressed as a percentage of the total peak area. Results are presented as mean ± standard deviation (SD) of three independent biological replicates.

### 4.5. Antioxidant Activity

#### 4.5.1. ABTS Antioxidant Activity

ABTS antioxidant activity was evaluated using the modified method of Rubio et al. [79]. The ABTS solution was prepared by mixing 7 mM ABTS with 2.45 mM potassium persulfate in 50% ethanol (*v*/*v*), followed by overnight incubation at room temperature. The solution was diluted to an absorbance of 0.70 ± 0.005 at 734 nm. Then, 0.25 mL of the sample was mixed with 2.5 mL of ABTS solution. The percentage of scavenging activity (I%) was calculated using the following equation:

I% = [(A_control_ − A_sample_)/A_control_] × 100

Results were expressed as the 50% inhibitory concentration (IC_50_) in mg mL^−1^.

#### 4.5.2. DPPH Scavenging Capacity

DPPH radical scavenging was determined following Quiroga et al. [80]. Briefly, 100 µL of the sample was mixed with 800 µL of 10 µM DPPH solution and dark incubated for 30 min at room temperature. Absorbance was then measured at 517 nm, and the scavenging activity (I%) was calculated using the following equation:

I%= [(A_control_ − A_sample_)/A_control_] × 100

Results were expressed as IC_50_ in mg mL^−1^.

#### 4.5.3. Ferric Reducing Antioxidant Power (FRAP)

The ferric reducing power assay, based on the ability to reduce Fe3+ to Fe2+, was performed according to Berker et al. [81]. Sample aliquots were mixed with 500 µL of phosphate buffer (0.2 M, pH 6.6) and 500 µL of potassium ferricyanide, then incubated at 50 °C for 30 min. After adding 500 µL of 10% triochloroacetic acid, the mixture was centrifuged at 3000 rpm for 10 min. The supernatant was added with 500 µL of distilled water and 100 µL of 0.1% FeCl_3_ and left at room temperature for 10 min. Absorbance was then measured at 700 nm. Reducing power was expressed as IC_50_ (mg mL^−1^), representing the extract concentration required to achieve an absorbance of 0.5.

#### 4.5.4. Total Antioxidant Activity (TAC)

Total antioxidant capacity (TAC) was determined following Rubio et al. [79], based on the reduction of phosphomolybdic acid to a phosphomolybdenum blue complex. The reagent consisted of 10 mL of sulfuric acid, 49 mg of ammonium molybdate, 72 mg of sodium phosphate, and 10 mL of distilled water. A 100 µL aliquot of the sample (1 mg sample in 1 mL ethanol) was mixed with 1 mL of the reagent. The mixture was incubated at 95 °C for 90 min, cooled to room temperature, and the absorbance (A) was measured at 695 nm. TAC was calculated using the appropriate formula.

TAC (%) = [(A_control_ − A_sample_)/A_control_] × 100

Results were expressed as IC_50_ (mg mL^−1^).

For all antioxidant assays (ABTS, DPPH, FRAP, and TAC), the three individual plant-derived EO samples per site were maintained separately, diluted in absolute ethanol, and evaluated at concentrations ranging from 0.02 to 2 mg mL^−1^. Each plant-derived EO constituted one biological replicate (*n* = 3 per site); any repeated assay readings for the same EO were technical replicates and were averaged before comparisons between sites.

### 4.6. Antifungal Assay

Antifungal activity against *Fusarium oxysporum* was evaluated by an essential-oil disc assay based on inhibition of mycelial growth [82] on PDA medium. For each of the three independent EO samples (Section 4.4.1), sterile Whatman paper discs were loaded with 10 µL of EO, and plates were prepared in triplicate (three plates per EO sample), giving nine plates per tested concentration in total (*n* = 3 independent EO samples, each averaged from 3 technical replicate plates). A 0.5 cm diameter mycelial plug was placed at the center of each plate. EO stock solutions were prepared in DMSO, and serial dilutions were prepared to obtain final EO concentrations ranging from 0.01 to 5 mg mL^−1^. The final DMSO concentration did not exceed 1% (*v*/*v*). PDA plates containing the corresponding amount of DMSO without EO served as untreated controls. Plates were incubated at 28 °C for 5 days in triplicate.

### 4.7. Antibacterial Activity Assay

The antibacterial activity of *T. satureioides* essential oil (EO) was evaluated using the broth microdilution method in 96-well microplates against the Gram-positive bacteria *Staphylococcus aureus* CIP 53154 and *Enterococcus hirae* CIP 5855, and the Gram-negative bacteria *Escherichia coli* CIP 54127 and *Pseudomonas aeruginosa* CIP A22. Minimum inhibitory concentrations (MICs) were determined according to the broth microdilution procedure described by the National Committee for Clinical Laboratory Standards (NCCLS) guidelines [83], with modifications adapted to the evaluation of essential oils.

Briefly, bacterial suspensions were prepared from fresh cultures and adjusted to approximately 10^8^ CFU mL^−1^. The EO was initially dissolved in dimethyl sulfoxide (DMSO) to prepare stock solutions, and serial two-fold dilutions were subsequently prepared in nutrient broth to obtain final EO concentrations ranging from 0.01 to 5 mg mL^−1^. The final concentration of DMSO in the assay did not exceed 1% (*v*/*v*). The bacterial suspensions were then added to the wells containing the different EO concentrations. Wells containing bacterial inoculum without EO served as growth controls, whereas wells containing nutrient broth without bacterial inoculum served as sterility controls. The microplates were incubated at 37 °C for 24 h. The MIC was defined as the lowest EO concentration at which no visible bacterial growth was observed.

### 4.8. Nematicidal Activity Assay

*Meloidogyne javanica* was isolated from infected tomato roots in Belfaa (Morocco), identified via perineal patterns, and maintained on tomato plants under greenhouse conditions. Egg masses were manually extracted from galled roots, surface-sterilized with 0.5% NaOCl for 1 min, and rinsed thrice with sterile distilled water [84]. Second-stage juveniles (J2s) were hatched, standardized to 80 J2s per 200 μL and stored at 4 °C. Nematicidal activity was evaluated by direct-contact method and the required amounts of EOs were first combined with Tween 80 at a 1:2 ratio, serving as an emulsifying agent, and then diluted with sterile distilled water (SDW) to obtain final concentrations (5, 10, and 15 µL. mL^−1^). A 2% Tween 80 solution and sterile distilled water were used as control treatments.

Egg hatch and juvenile mortality were assessed daily. Observations of juvenile mortality were continued until complete mortality (100%) of the hatched juveniles was achieved. Each concentration (5, 10, and 15 µL mL^−1^) was tested in three independent biological replicates (*n* = 3). Each replicate consisted of 80 J2 juveniles for the juvenile mortality assay and 14 eggs for the egg-hatching assay.

### 4.9. Statistical Analysis

Statistical analyses were performed using R software version 4.4.3. Data are expressed as mean ± standard error of three replicates (*n* = 3). Treatment differences were assessed by analysis of variance (ANOVA) followed by Tukey’s Honestly Significant Difference (HSD) test for multiple comparisons (*p* < 0.05). Means followed by different letters were considered significantly different.

## 5. Conclusions

This study demonstrates that, under the specific wild population and cultivated site investigated, cultivation was associated with changes in essential oil (EO) production, chemical composition, biological activities, and phospholipid fatty acid (PLFA)-based microbial profiles in *T. satureioides*. The observed differences between wild and cultivated plants highlight the potential influence of cultivation conditions on EO quality and bioactive properties. These findings suggest that cultivation practices should focus not only on plant growth and yield but also on maintaining soil properties and microbial functions that may contribute to secondary metabolite production. Accordingly, improved soil management and reduced soil disturbance may help preserve desirable EO characteristics in cultivated plants while promoting sustainable production and reducing harvesting pressure on wild populations. However, the findings are limited to the specific wild population and cultivated site investigated, and further research is needed to validate these observations across different environments. Replicated multi-site studies incorporating plant-specific EO chemical analyses and matched environmental measurements are required to identify reliable agronomic practices that preserve desirable EO characteristics under cultivation conditions. Such studies will help establish cultivation strategies that support both EO quality and sustainable production while reducing pressure on natural populations.

## Figures and Tables

**Table 1:** Soil physicochemical properties of wild and cultivated *T. satureioides* sites. Values are expressed as mean ± standard deviation (SD) (*n* = 3). Differences between the two sites were evaluated using one-way analysis of variance (ANOVA). Different superscript letters indicate statistically significant differences at *p* < 0.05.

Parameters	WT	CT
Organic carbon (g kg^−1^)	0.31 ± 0.04 ^a^	1.31 ± 0.59 ^b^
Organic matter (g kg^−1^)	0.54 ± 0.09 ^a^	2.91 ± 0.65 ^b^
Nitrogen (g kg^−1^)	3.03 ± 0.87 ^a^	3.98 ± 0.54 ^a^
Total Phosphorus (g kg^−1^)	1.11 ± 0.08 ^a^	1.94 ± 0.05 ^b^
Total limestone (g kg^−1^)	36.41 ± 1.16 ^a^	41.94 ± 5.76 ^a^
pH ^water^	7.83 ± 0.35 ^a^	7.97 ± 0.30 ^a^
pH ^KCl^	6.43 ± 0.13 ^a^	7.06 ± 0.44 ^a^
Conductivity (us cm^−1^)	48.06 ± 9.25 ^a^	113.16 ± 13.16 ^b^
Humidity (%)	6.12 ± 0.71 ^a^	25.82 ± 1.64 ^b^

**Table 2:** Soil composition of wild and cultivated *T. satureiodes* soil sites using XRF.

%	C (SiO_2_)	C (TiO_2_)	C (Al_2_O_3_)	C (Fe_2_O_3_)	C (MnO)	C (MgO)	C (CaO)	C (Na_2_O)	C (K_2_O)	Sum of Oxides Plus Loss on Ignition
WT	27.34	0.776	2.393	5.133	0.111	1.306	14.711	2.597	1.506	77.979
CT	25.31	0.732	2.665	5.381	0.110	1.047	18.375	0.226	1.590	79.567

**Table 3:** EO yield of wild (WT) and cultivated (CT) *T. satureioides*.

Treatment	EO Yield (%)
Cultivated (CT)	1.60 ± 0.09 ^a^
Wild (WT)	1.00 ± 0.12 ^b^

Different lowercase letters (a, b) indicate statistically significant differences between WT and CT (*p* < 0.05).

**Table 4:** Chemical composition of EO from cultivated (CT) and wild (WT) *T. satureioides*. Relative abundances of volatile compounds are expressed as percentage (%) of the total GC–MS peak area.

Name	RT_min_	RI_exp_	RII_it_	RI_NIST_	WT (%)	CT (%)
α-Thujene	2.122	923	917	925	--	1.01 ± 0.05
α-Pinene	2.176	939	922	939	4.29 ± 0.20 ^a^	4.20 ± 0.35 ^a^
Camphene	2.285	953	933	953	6.54 ± 0.41 ^a^	6.13 ± 0.10 ^a^
β-Pinene	2.444	981	949	981	0.67 ± 0.03 ^a^	0.70 ± 0.04 ^a^
β-Myrcene	2.487	991	953	991	--	0.64 ± 0.03
4-Carene	2.67	1001	1013	1004	0.79 ± 0.42 ^a^	1.08 ± 0.004 ^a^
p-Cymene	2.717	1030	1030	1028	5.63 ± 0.33 ^a^	5.23 ± 0.26 ^a^
Limonene	2.745	1034	1036	1031	5.30 ± 0.24 ^a^	1.45 ± 0.03 ^b^
γ-Terpinene	2.918	1059	1038	1060	7.39 ± 0.19 ^a^	7.52 ± 0.07 ^a^
α-Terpinolene	3.094	1084	1056	1083	--	0.13 ± 0.01
Linalool	3.151	1098	1101	1098	2.13 ± 0.04 ^b^	3.21 ± 0.13 ^a^
Camphor	3.483	1143	1159	1143	0.28 ± 0.04	--
endo-Borneol	3.647	1165	1151	1165	16.37 ± 0.58 ^a^	15.61 ± 0.81 ^a^
Terpinen-4-ol	3.674	1177	1154	1177	--	1.67 ± 0.09
α-Terpineol	3.754	1207	1203	1200	8.23 ± 0.77 ^a^	5.79 ± 0.08 ^b^
Isobornyl formate	3.943	1238	1233	1227	0.38 ± 0.03 ^a^	0.06 ± 0.01 ^b^
Thymol methyl ether	3.969	1243	1224	1235	--	4.49 ± 0.5
Thymol	4.276	1290	1291	1290	24.79 ± 0.65 ^b^	31.83 ± 1.15 ^a^
Carvacrol	4.295	1301	1301	1300	--	2.30 ± 0.23
α-Copaene	4.75	1376	1346	1376	0.20 ± 0.01 ^a^	0.23 ± 0.04 ^a^
Caryophyllene	5	1428	1418	1429	--	4.54 ± 0.04
α-Humulene	5.176	1440	1436	1438	0.20 ± 0.01 ^a^	0.21 ± 0.04 ^a^
δ-Cadinene	5.455	1527	1513	1524	0.40 ± 0.08 ^b^	0.82 ± 0.02 ^a^
Caryophyllene oxide	5.804	1573	1548	1575	0.53 ± 0.04 ^a^	0.48 ± 0.03 ^a^
T-Muurolol	6.054	1653	1653	1647	1.06 ± 0.03 ^a^	0.63 ± 0.02 ^b^
hydrocarbon monoterpenes					30.61	28.09
oxygenated monoterpenes					52.18	64.96
hydrocarbon sesquiterpenes					0.8	5.80
oxygenated sesquiterpenes					1.59	1.11
Total percentage %					85.18	99.96

Footnote: RT, retention time; RIexp, experimental retention indices determined relative to an n-alkane series (C8–C40) on a ZB-5MS capillary column; RILit, retention indices obtained from literature and the Pherobase Database of Pheromones and Semiochemicals; RINIST, retention indices obtained from the NIST23 Database (version 23). Relative percentages (%) represent the relative abundance of compounds identified by GC-MS analysis and are expressed as mean ± standard deviation of three independent oil samples (*n* = 3). Different lowercase letters within the same row indicate significant differences between WT-EO and CT-EO (one-way ANOVA, *p* < 0.05; without adjustment for multiple testing across compounds).

**Table 5:** Antimicrobial activity of wild and cultivated *T. satureioides* essential oils expressed as minimum inhibitory concentrations (MICs). Different superscript letters (a, b) indicate statistically significant differences at *p* < 0.05.

	WT-EO MIC (mg mL^−1^)	CT-EO MIC (mg mL^−1^)
***Escherichia coli***	2.50 ± 0.15 ^b^	3.80 ± 0.66 ^a^
***Pseudomonas aeruginosa***	2.50 ± 0.14 ^b^	3.80 ± 0.45 ^a^
***Enterococcus hirae***	0.05 ± 0.001 ^b^	0.10 ± 0.001 ^a^
***Staphylococcus aureus***	0.04 ± 0.003 ^b^	0.50 ± 0.06 ^a^
***Fusarium oxysporum***	0.155 ± 0.09 ^b^	0.187 ± 0.08 ^a^

## Data Availability

The original contributions presented in this study are included in the article and its Supplementary Materials. Further inquiries can be directed to the corresponding authors.

## Supplementary Materials

The following supporting information can be downloaded at: https://www.mdpi.com/article/10.3390/plants15213398/s1, Figure S1: GC–MS chromatogram of the essential oil from cultivated *Thymus satureioides*; Figure S2: GC–MS chromatogram of the essential oil from cultivated *Thymus satureioides*.
