# Supplementary material for: Comparison of Wild and Field-Cultivated Thymus satureioides: Effects on Rhizosphere Properties, Essential-Oil Composition, and Biological Activities
Source: Plants (Basel). 2026 Nov 2;15(21):3398. doi: 10.3390/plants15213398 (PMC13766068; doi:10.3390/plants15213398)
Supplement: Supplementary file 1 [file plants-15-03398-s001.zip › plants-4525518-supplementary.pdf]

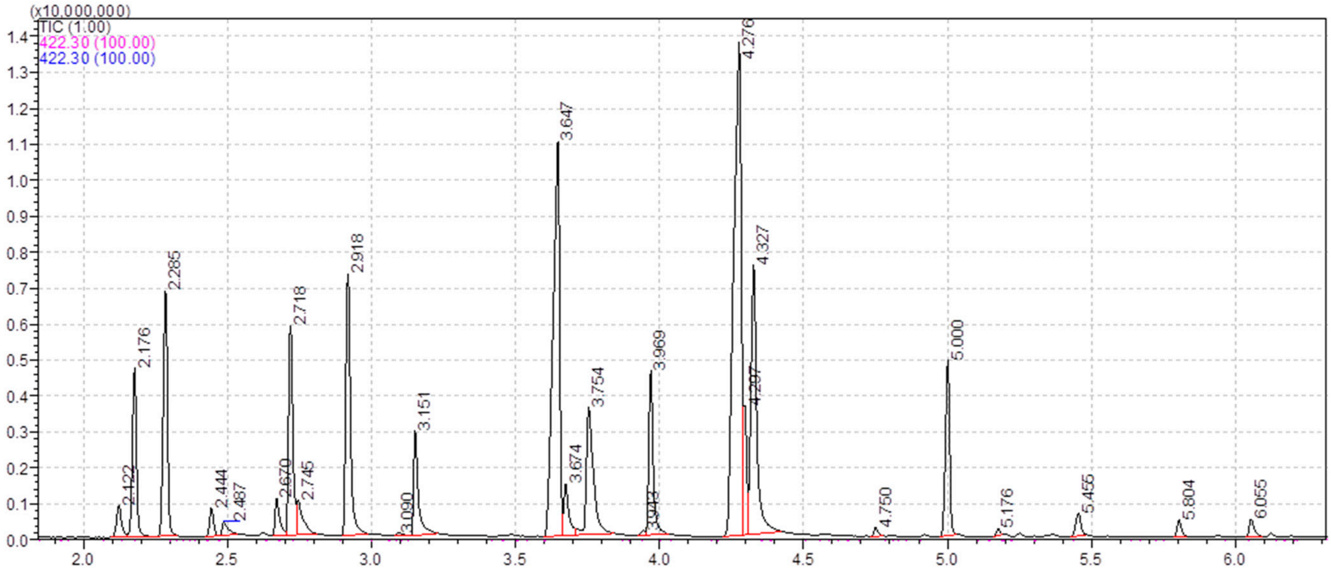

Figure S1 : GC–MS chromatogram of the essential oil from cultivated *Thymus satureioides*

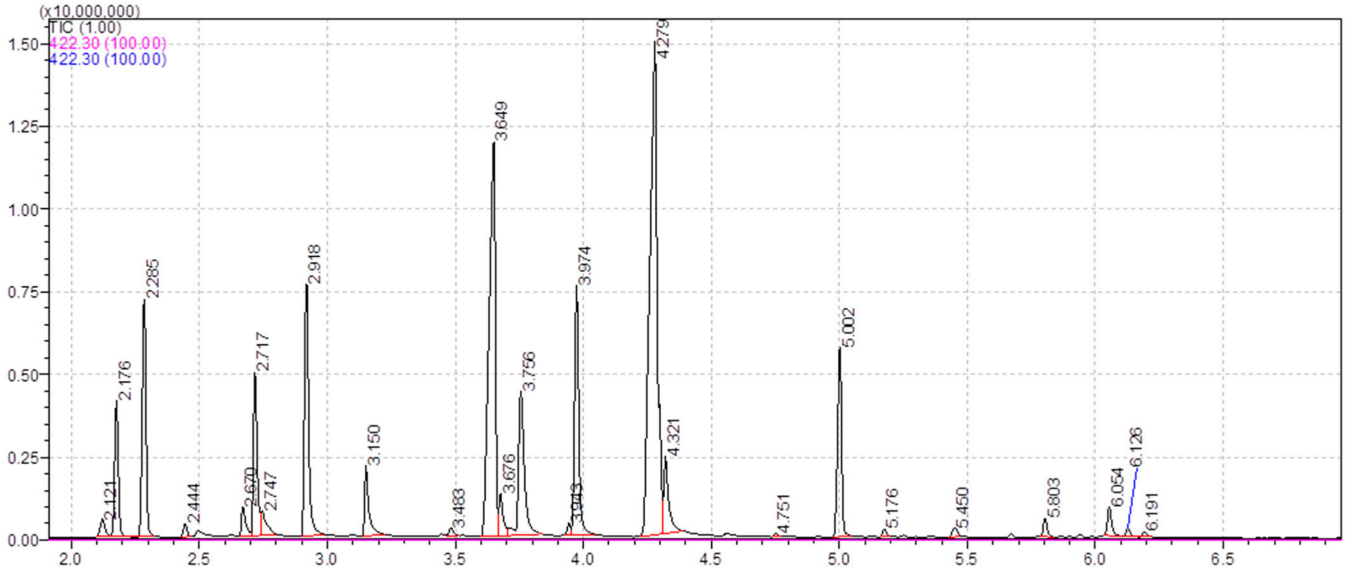

Figure S2 : GC–MS chromatogram of the essential oil from cultivated *Thymus satureioides*
